# Nur77-mediated TRAF6 signalling protects against LPS-induced sepsis in mice

**DOI:** 10.1186/s12950-016-0112-9

**Published:** 2016-02-02

**Authors:** Xiu-Ming Li, Shen Zhang, Xiao-Shun He, Peng-Da Guo, Xing-Xing Lu, Jing-Ru Wang, Jian-Ming Li, Hua Wu

**Affiliations:** Pathology Center and Department of Pathology, Soochow University, Suzhou, 215123 China; The First Affiliated Hospital of Soochow University, Suzhou, 215006 China

**Keywords:** Nur77, Inflammation, Sepsis, LPS, Animal study

## Abstract

**Background:**

Nur77, a key member of the NR4A receptor subfamily, is involved in the regulation of inflammation and immunity. However, the in vivo regulatory roles of Nur77 in sepsis and the mechanisms involved remains largely elusive. In this study, we used Nur77-deficient (Nur77^−/−^) mice and investigated the function of Nur77 in sepsis.

**Findings:**

Compared to wild-type (Nur77^+/+^) mice, Nur77^−/−^ mice are more susceptible to LPS-induced sepsis and acute liver inflammation. Mechanistically, we observed that Nur77 can interact with TRAF6, a crucial adaptor molecule in the Toll-like receptor-interleukin 1 receptor (TLR-IL-1R) signalling pathway, in in vivo mouse model of sepsis. The interaction may affect TRAF6 auto-ubiquitination, thereby inhibiting NF-κB activation and pro-inflammatory cytokines production.

**Conclusions:**

These in vivo observations reveals an important protective role for Nur77 in LPS-induced sepsis through its regulation to TRAF6 signalling, and highlights the potential clinical application of Nur77 as a molecular target in prevention and/or treatment of sepsis.

**Electronic supplementary material:**

The online version of this article (doi:10.1186/s12950-016-0112-9) contains supplementary material, which is available to authorized users.

## Introduction

Orphan nuclear receptor Nur77 (also called TR3, NGFI-B, or NR4A1) is a member of the NR4A family of nuclear receptors. Similar to other nuclear receptors, Nur77 consists of an N-terminal transactivation domain, a central DNA binding domain and a C-terminal ligand binding domain, and can act in the nucleus as a ligand-independent and constitutively active transcription factor by binding to its DNA response elements as monomers [1], homodimers [2] or heterodimers with retinoid X receptor [2]. Unlike other nuclear receptors, Nur77 and other members of the subfamily are classified as early response genes whose expression is induced by a diverse range of extracellular stimuli including a wide array of cytokines and growth factors [3]. Consistently, accumulating studies indicate that Nur77 is implicated in the control of inflammatory diseases including atherosclerosis [4], arthritis [5], inflammatory bowel disease (IBD) [6] and cancer [7]. Nur77 is aberrantly expressed in atherosclerotic lesions [8], and cancer [7]. Nur77 may act to mediate pro-inflammatory signalling by increasing the expression of NF-κB-activating kinase, IKKi [9] to attenuate cytokine signalling. However, recent in vivo studies have shown that Nur77 is protective against the development of atherosclerosis by regulating the polarization of macrophages and subsequently inhibits inflammatory responses [10], indicating Nur77 may mediate anti-inflammatory signalling. Despite all of these efforts, however, the in vivo roles and underlying mechanism of Nur77 in sepsis are unclear.

Tumor necrosis factor receptor associated factor 6 (TRAF6), a member of TRAF family, is a common signalling mediator for the TLR-IL-1R superfamily [11]. TRAF6-deficient mice have defects in TLR-IL-1R-initiated inflammatory signalling [12]. TRAF6 is reported to possess an E3 ubiquitin ligase and undergoes lysine 63 (K63)-linked auto-ubiquitination [13]. The modification, in contrast to K48-linked polyubiquitin conjugation, is not associated with proteasomal degradation but instead facilitates signal transduction and protein trafficking [14, 15]. The regulatory involvement of various molecules in TRAF6-mediated TLR-IL-1R signalling has been confirmed. β-arrestin act as negative regulators to prevent ubiquitination of TRAF6 through direct interaction, which effectively blocks excessive inflammatory responses [16]. In contrast, the formation of TRAF6-HSP27 complex promotes TRAF6 ubiquitination and enhances activation of NF-κB signalling triggered by IL-1β [17].

Here, we investigate the in vivo function of Nur77 in sepsis and sepsis-associated liver injury. Our work indicates that Nur77 deficiency in mice increased their susceptibility to LPS-induced sepsis and acute liver injury, and reveal a critical mechanism wherein Nur77 interacts with TRAF6 and regulate its auto-ubiquitination in in vivo mouse model of sepsis.

## Materials and methods

### Mice

Nur77-knockout mice were purchased from Jackson Laboratory (Bar Harbor, ME). Mice were maintained in a pathogen-free environment as recently described [6]. All animal experiments were performed in accordance with the regulations and guidelines of the Animal Care and Use Committee of Soochow University.

### Sepsis model

The age- and sex-matched Nur77^+/+^ versus Nur77^−/−^ mice were injected with LPS (20 mg/kg, ip). Then, 2 h later, hepatic *Tnf*, *Il6,* and *Il12b* mRNA was measured by RT-PCR and Real-time PCR. TNFα and IL-6 in blood were measured with ELISA. To induce endotoxic shock, mice were injected with LPS (20 mg/kg, ip) and were monitored for survival for the ensuing 72 h.

### Acute liver injury model

Nur77^+/+^ and Nur77^−/−^ mice used for the model were 8–10 weeks of age and were matched for age and sex. Mice were co-injected with LPS (5 μg/kg, ip) and D-GalN (400 mg/kg). 5 h later, mice were anesthetized with ether and retro-orbitally bled. ALT, AST, TNFα, and IL-6 were measured with ELISA. Also, RNA was extracted from liver tissue and relative mRNA of *Tnf*, *Il6,* and *Il12b* were measured by RT-PCR and Real-time PCR. Mice were monitored for 24 h to assess survival.

### Tissue samples collection and evaluation

Pathological analysis of lung, liver and kidney from Nur77^+/+^ and Nur77^−/−^ mice was conducted and tissue samples were fixed in 10 % buffered formalin and then embedded in paraffin. Tissue was sectioned and stained with hematoxylin and eosin (H&E) according to standard histological procedures.

### Real-time PCR assays

Real-time PCR assays were performed as published [7, 18]. The abundance of each mRNA was normalized relative to PCR with the housekeeping gene β-actin. The primers for PCR reactions are listed in Table 1.

**Table 1:** Primers for real-time PCR

Mouse gene name	Forward/reverse
*Tnf*	F: 5’-CTCACACTCAGATCATCTTCTC-3’
	R: 5’-CTTTCTCCTGGTATGAGATAGC-3’
*Il6*	F: 5’-TTCCATCCAGTTGCCTTCTTG-3’
	R: 5’-AGGTCTGTTGGGAGTGGTATC-3’
*Il12*	F: 5’- CAACATCAAGAGCAGTAGCAG-3’
	R: 5’- TACTCCCAGCTGACCTCCAC-3’
*β-actin*	F: 5’-TGGAATCCTGTGGCATCCATGAAAC-3’
	R: 5’-TAAAACGCAGCTCAGTAACAGTCCG-3’

### ELISA

ALT, AST, TNFα, and IL-6 in serum were measured with commercially available kits according to the manufacturer’s instructions.

### Western blot

Western blot analyses were performed as described in the literature [7].

### Immunoprecipitation and ubiquitination Assay

Cells were lysed in lysis buffer (2 mmol/L Tris–HCl (pH 7.4), 10 mmol/L EDTA, 100 mmol/L NaCl and 1 % IGEPAL). Cell lysates were incubated with indicated antibodies in protein A/G beads (Santa Cruz Biotechnology) for 3 h. Then, the protein-antibody complexes on the beads were analyzed with Western blot. To measure TRAF6 ubiquitination, 10 mM N-ethylmaleimide (Sigma) was included in the lysis buffer.

### Statistical analysis

Data are expressed as means ± SD, and Student’s *t* test (unpaired, two-tailed) was used to compare two groups of independent samples (*p* < 0.05 were considered statistically significant).

## Results and discussion

**Fig. 1:**
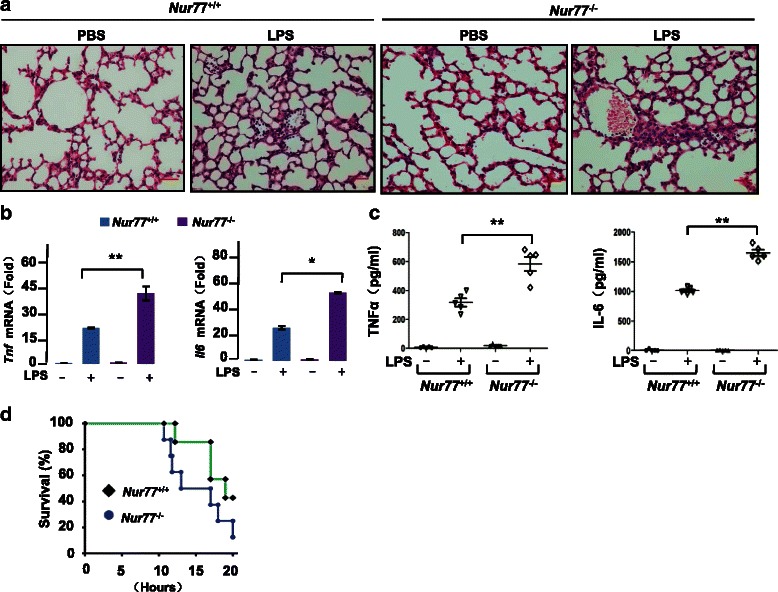
Mice deficient in Nur77 are more susceptible to LPS-induced sepsis. **a** Histological analysis of lungs from Nur77^+/+^ and Nur77^−/−^ mice 6 h after challenge with PBS or LPS (20 mg/kg). Representative images are shown. Scale bars, 100 μM. Original magnification, 100x. **b** RT-PCR (left) and Real-time PCR (right) quantification of expression of hepatic *Tnf* and *Il6* mRNA from Nur77^+/+^ and Nur77^−/−^ mice 2 h after challenge with PBS or LPS (20 mg/kg). Error bars represent means ± SD from 3 biological replicates. *p < 0.05 and **p < 0.01. **c** ELISA assay of TNFα and IL-6 expression in serum of LPS-treated Nur77^+/+^ mice (n = 5) and LPS-treated Nur77^−/−^ mice (n = 5). Error bars represent means ± SD. **p < 0.01. **d** Survival of Nur77^+/+^ mice (n = 7) and Nur77^−/−^ mice (n = 8) treated with LPS and monitored for up to 24 h

Research suggests that orphan nuclear receptor Nur77 is implicated in inflammation and immunity. Mice lacking all Nr4a receptors including Nur77 did not generate T_reg_ cells and resulted in systemic autoimmune disease [19]. Nur77 deficiency in mice lead to acceleration of atherosclerosis [10] and inflammatory bowel disease [6]. We recently reported that loss of Nur77 in older mice contributes to systemic inflammation [20]. Sepsis, the major complication of severe infection, usually causes multisystem organ failure and even death of many patients in hospital [21]. Therefore, it is of great importance to find the new potential targets for sepsis treatment. Here we investigate in vivo functions of Nur77 in sepsis and sepsis-associated liver injury. We first challenged Nur77^−/−^ mice with LPS to ascertain the role of Nur77 in LPS-induced sepsis in vivo. After treatment with LPS, lungs of Nur77^−/−^ mice had severe inflammatory hyperemia, as evidenced by increased mononuclear cells and erythrocyte infiltration (Fig. 1a). RT-PCR and Real-time PCR assays, in liver tissues from Nur77^−/−^ mice, showed substantial induction of *Tnf* and *Il6* expression (Fig. 1b). Consistent with these results, levels of TNFα and IL-6 in serum were significantly higher in LPS-treated Nur77^−/−^ mice than in LPS-treated Nur77^+/+^ mice (Fig. 1c), suggesting that the host response in Nur77^−/−^ mice is altered. After lethal challenge with LPS, Nur77^−/−^ mouse survival was reduced (Fig. 1d). These results are consistent with a recent report that mice lacking Nur77 had exacerbated inflammatory and immune responses, and survival was decreased after lethal endotoxemic challenge [22]. Thus, Nur77 is important for modulation of inflammatory responses during sepsis.

**Fig. 2:**
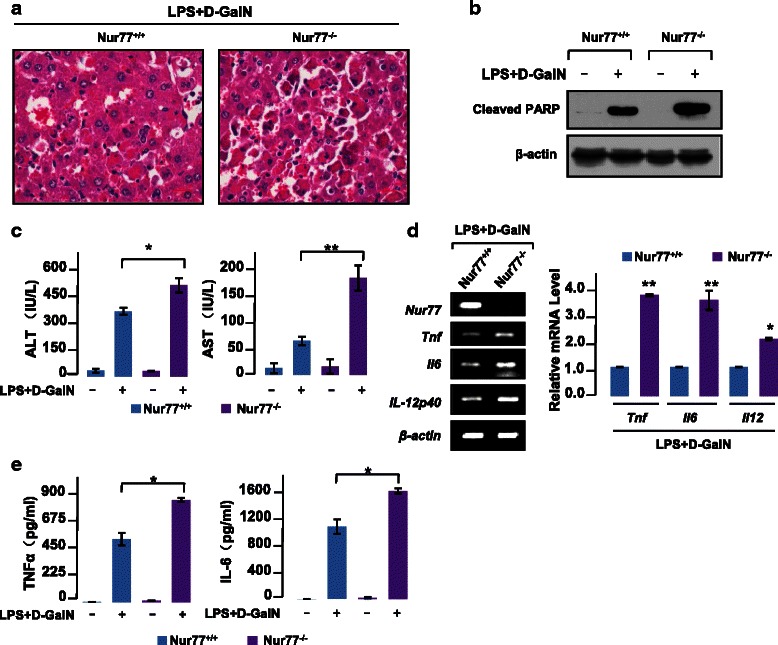
Nur77^−/−^ mice have increased susceptibility to LPS/D-GalN-induced acute liver injury. **a** H&E staining of livers from Nur77^+/+^ and Nur77^−/−^ mice 5 h after injection with LPS/D-GalN. Representative images are shown. Scale bars, 100 μM. Original magnification, 100x. **b** Liver extracts assessed by Western blot and indicated antibodies. **c** ELISA of serum ALT and AST from Nur77^+/+^ and Nur77^−/−^ mice 5 h after injection with LPS/D-GalN or PBS. Error bars represent means ± SD. *p < 0.05 and **p < 0.01. **d** *Tnf*, *Il6* and *Il12* mRNA expression was measured with RT-PCR (left) and qPCR (right) in livers from Nur77^+/+^ and Nur77^−/−^ mice 2 h after treatment with LPS/D-GalN. Error bars represent means ± SD from 3 biological replicates. *p < 0.05 and **p < 0.01. **e** ELISA quantification of serum TNFα, IL-6 from Nur77^+/+^ and Nur77^−/−^ mice after treatment with LPS/D-GalN for 5 h. Error bars represent means ± SD. *p < 0.05

To further ascertain the role of Nur77 in sepsis, we treated mice with LPS/D-GalN known to induce acute liver injury. At 5 h after LPS/D-GalN challenge, Nur77^−/−^ mice had severe hepatocyte destruction compared to wild-type mice (Fig. 2a). LPS/D-GalN injection also promoted hepatocyte cell death in Nur77^−/−^ mice revealed by PARP cleavage (Fig. 2b). ALT and AST, liver function markers, were also significantly greater in serum from LPS/D-GalN-treated Nur77^−/−^ mice (Fig. 2c), indicating increased liver necrosis. We also measured TNFα and IL-6 expression as these are known to be involved in this model of acute liver inflammation [23, 24] in liver tissues from wild-type and Nur77^−/−^ mice. RT-PCR and real-time PCR assays confirmed that expression of these pro-inflammatory cytokines mRNA was greater in LPS/D-GalN-treated Nur77^−/−^ mice than in wild-type mice (Fig. 2d). Similarly, generation of inflammatory cytokines including TNFα and IL-6 were markedly enhanced in serum from Nur77^−/−^ mice (Fig. 2e). Thus, Nur77 is protective against LPS-induced acute liver injury.

**Fig. 3:**
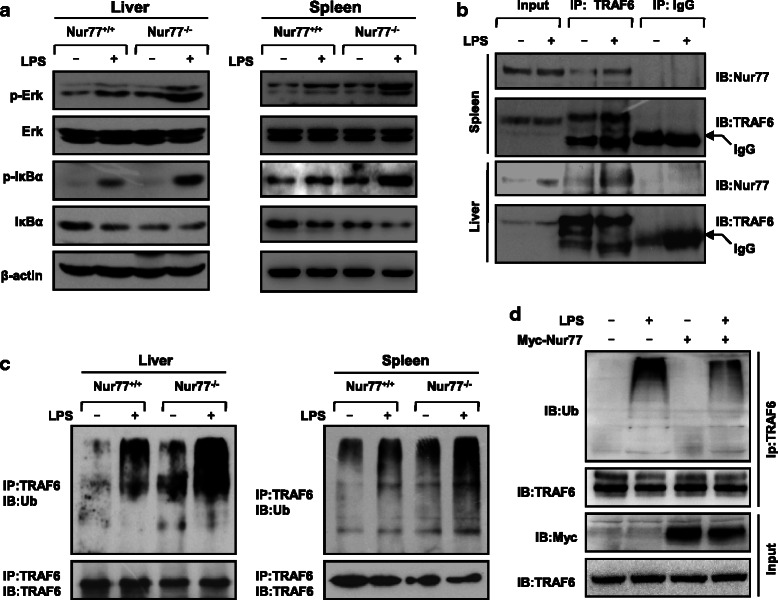
Nur77 inhibits inflammatory response in in vivo mouse model of sepsis by interacting with TRAF6 and regulating TRAF6 auto-ubiquitination (**a**) Immunoblot analysis of indicated signalling proteins in liver (left) and spleen (right) from Nur77^+/+^ and Nur77^−/−^ mice 2 h after LPS (20 mg/kg) challenge. **b** Immunoprecipitation and immunoblot of liver and spleen proteins from C57BL/6 mice 1 h after LPS (20 mg/kg) treatment. **c** Immunoprecipitation of endogenous TRAF6 from liver (left) and spleen (right) lysates of LPS-treated Nur77^+/+^ and Nur77^−/−^ mice, and immunoblot of TRAF6 auto-ubiquitination with anti-ubiquitin antibody. **d** Immunoprecipitation of endogenous TRAF6 from lysates of LPS-treated (50 ng/ml) RAW264.7 cells expressing vector or myc-tagged Nur77 plasmid and immunoblotted for TRAF6 auto-ubiquitination with an anti-ubiquitin antibody

The molecular mechanism by which Nur77 deficiency promotes sepsis remains obscure. Nur77 has been shown to inhibit LPS-induced inflammation by inhibiting p65 binding to DNA, thereby reducing pro-inflammatory cytokine production [25]. We observed enhanced phosphorylation and degradation of IκBα was in liver and spleen tissues from Nur77^−/−^ mice challenged with LPS (Fig. 3a), indicating Nur77 could suppress LPS-induced NF-κB activity in vivo. Also Nur77 contributes to regulation of TRAF6 signalling through its interaction with TRAF6. As shown in Fig. 3b, mice challenged with LPS for 1 h had enhanced Nur77-TRAF6 interaction in the liver and spleen compared to control PBS-treated mice. These results are consistent with our recent observation that disruption of Nur77-TRAF6 interaction in Nur77^−/−^ mice accelerated the development of IBD [6]. Collectively, these data suggest that Nur77 physically interacts with TRAF6 in in vivo mouse model of sepsis, revealing a pathophysiological significance of Nur77-TRAF6 interaction in sepsis. Auto-ubiquitination of TRAF6 is required for NF-κB signal transduction [16]. Here, our results showed that Nur77 deficiency significantly enhanced auto-ubiquitination of TRAF6 in liver and spleen tissues prepared from Nur77^−/−^ but not wild-type mice (Fig. 3c). At the same time, overexpression of Nur77 significantly impaired LPS-induced TRAF6 auto-ubiquitination (Fig. 3d). Also we investigated whether Nur77 can affect auto-ubiquitination of TRAF3, another member of the TRAF family but we observed no significant change. Additional file 1: Figure S1 indicates that overexpression of Nur77 did not affect TRAF3 auto-ubiquitination induced by LPS, suggesting that Nur77 is important in regulating LPS-induced inflammation by targeting TRAF6.

In summary, our in vivo study confirmed a critical protective role for the orphan nuclear receptor Nur77 in sepsis and identify a key mechanism for Nur77 in the regulation of TRAF6 signalling through its interaction with TRAF6 in in vivo mouse model of sepsis.

## Additional file

Additional file 1: Figure S1. Nur77 does not affect auto-ubiquitination of TRAF3 induced by LPS. Immunoprecipitation of endogenous TRAF3 from lysates of LPS-treated (50 ng/ml) RAW264.7 cells expressing vector or myc-tagged Nur77 plasmid, immunoblotted for TRAF3 auto-ubiquitination with an anti-ubiquitin antibody. (PDF 62 kb)

## Abbreviations

ELISA: Enzyme Linked Immunosorbent Assay
TNFα: Tumor Necrosis Factor alpha
IL-6: Interleukin-6
IL-12: Interleukin-12
