# Supplementary figures and images for: Nur77-mediated TRAF6 signalling protects against LPS-induced sepsis in mice
Source: J Inflamm (Lond). 2016 Feb 2;13:4. doi: 10.1186/s12950-016-0112-9 (PMC4735956; doi:10.1186/s12950-016-0112-9)

# Supplementary Figure 1

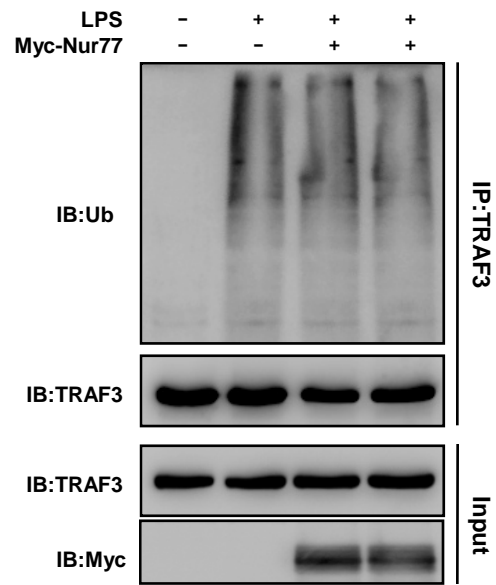

Supplement: Additional file 1: Figure S1. — Nur77 does not affect auto-ubiquitination of TRAF3 induced by LPS. Immunoprecipitation of endogenous TRAF3 from lysates of LPS-treated (50 ng/ml) RAW264.7 cells expressing vector or myc-tagged Nur77 plasmid, immunoblotted for TRAF3 auto-ubiquitination with an anti-ubiquitin antibody. (PDF 62 kb) [file 12950_2016_112_MOESM1_ESM.pdf]
